# Modeling the Stiffness of Coupled and Uncoupled Recycled Cotton Fibers Reinforced Polypropylene Composites

**DOI:** 10.3390/polym11101725

**Published:** 2019-10-21

**Authors:** Albert Serra, Quim Tarrés, Miquel-Àngel Chamorro, Jordi Soler, Pere Mutjé, Francesc X. Espinach, Fabiola Vilaseca

**Affiliations:** 1 LEPAMAP Group, Department of Chemical Engineering, University of Girona, 17003 Girona, Spain; albert.serra@udg.edu (A.S.); joaquimagusti.tarres@udg.edu (Q.T.); pere.mutje@udg.edu (P.M.); 2 Càtedra de Processos Industrials Sostenibles, University of Girona, 17003 Girona, Spain; 3 Department of Architecture and Construction, 17003 Girona, Spain; mangel.chamorro@udg.edu (M.-À.C.); jordi.soler@udg.es (J.S.); 4 Design, Development and Product Innovation, Dept. of Organization, Business, University of Girona, 17003 Girona, Spain; 5 Advanced Biomaterials and Nanotechnology, Dpt. of Chemical Engineering, University of Girona, 17003 Girona, Spain; fabiola.vilaseca@udg.edu

**Keywords:** recycled cotton fibers, stiffness, micromechanics, Young’s modulus

## Abstract

The stiffness of a composite material is mainly affected by the nature of its phases and its contents, the dispersion of the reinforcement, as well as the morphology and mean orientation of such reinforcement. In this paper, recovered dyed cotton fibers from textile industry were used as reinforcement for a polypropylene matrix. The specific dye seems to decrease the hydrophilicity of the fibers and to increase its chemical compatibility with the matrix. The results showed a linear evolution of the Young’s moduli of the composites against the reinforcement contents, although the slope of the regression line was found to be lower than that for other natural strand reinforced polypropylene composites. This was blamed on a growing difficulty to disperse the reinforcements when its content increased. The micromechanics analysis returned a value for the intrinsic Young’s modulus of the cotton fibers that doubled previously published values. The use of two different micromechanics models allowed evaluating the impact of the morphology of the fibers on the Young’s modulus of a composite.

## 1. Introduction

The use of fibrous industrial byproducts as reinforcement for polymer-based composites has increasingly been attracting the attention of researchers. The use of byproducts is in line with the principles of green chemistry and the actual demands of the society for greener materials and more environmentally friendly products [1,2,3]. The literature shows the opportunity to use agroforestry wastes such as prunings, used paper fibers, or textile byproducts [4,5,6,7,8]. These studies reveal how the nature of the reinforcements has a high impact on the mechanical properties of its composites. In this sense, artificial fibers like glass fibers, aramids, or carbon fibers show the highest strengthening and stiffening abilities [9,10]. Natural fiber strands and wood fibers also show notable capabilities as polyolefin reinforcements. Nonetheless, strands like jute or hemp showed higher stiffening potential than wood fibers [11,12,13,14,15]. In this sense, cotton strands have been used as polyolefin reinforcement successfully [16,17,18]. While some of the studies have used raw cotton as reinforcing fibers, a vast majority prefer to use recycled fibers from the textile industry [7,13,19,20], however, the number of published studies are still limited.

Cotton fibers recovered from the textile industry have some advantages, such as low cost and availability, but also some apparent drawbacks, since usually these fibers contain textile dyes [7,20]. Additionally, there is a large quantity of discarded textiles that are directly landfilled [21]. Moreover, landfilled textiles contribute to the formation of ‘leachate’ that can contaminate ground and underground waters [22]. Thus, the use of such textiles as composite reinforcement can contribute to widen the value chain of the textile sector on the one hand, and to decrease landfilling and contamination on the other hand.

Cotton fibers are almost 100% cellulosic fibers, and thus have a high presence of hydroxyl groups in their surface, and a high potential to create hydrogen bonds under favorable conditions. Therefore, they tend to aggregate, making their individualization and dispersion on a polymeric phase difficult [8]. In addition, cotton fibers are hydrophilic, while the vast majority of polymeric matrices are hydrophobic.

A previous study revealed that the presence of dyes diminished the hydrophilicity of cotton fibers, allowing the obtaining of better interphases without using any coupling agent [7,20]. The same dyes eased the dispersion of the fibers without any treatment. Nonetheless, the tensile strength of the composites reinforced with dyed cotton fibers were lower than those obtained with other natural fiber strands. Some authors claim that the same dyes hindered the action of coupling agents [7], although these researchers did not publish any results concerning the stiffness of the composites. According to the literature, the intrinsic Young’s modulus of cotton fibers is found between 5 and 13 GPa, however, this value seems too low compared with the values obtained for other strands [11,12,23,24,25,26,27,28].

This paper examines the Young’s modulus of cotton fiber (CF) reinforced polypropylene (PP) composites. CFs were recovered from a yarning process where all the fibers with lengths below 10mm were discarded. The byproduct has the shape of cotton dyed flocks that must be individualized prior to its use as reinforcement [20]. Composite materials adding CF percentages ranging from 20 to 50 wt% were formulated. Two batches of every formulation were prepared, one with 6 wt% of coupling agent added, and the other without. The composites were mold injected to obtain the standard specimens, and later on tested under tensile conditions. The Young’s moduli of the materials were evaluated and discussed. The Young’s moduli of the composites were not coherent with the intrinsic Young’s modulus for CFs found in the literature. Therefore, a micromechanical analysis was proposed to analyze the properties of CF. First, the Hirsch model provided a value for the intrinsic Young’s modulus of CF that doubled those on the literature [29]. Then, the efficiency factors allowed discussing a possible poor dispersion of the fibers at high reinforcement contents. Finally, the Tsai and Pagano model in combination with Halpin and Tsai equations [30,31] allowed incorporating the morphology of the reinforcements to back-calculate a theoretical Young’s modulus for the composites. The paper actualizes the value of the intrinsic Young’s modulus of cotton fibers, and proposes a series of composites that reuse textile byproducts, and thus avoids their landfilling or incineration.

## 2. Materials and methods

### 2.1. Materials

The cotton fibers (CF) used as reinforcement were recovered from cotton flock residues. These cotton flocks are textile industry byproducts and are composed of entangled cotton fibers with lengths too short for spinning. The flocks were previously treated with a reactive dye and were kindly supplied by Fontfilva S. L. (Olot, Girona, Spain). 

A polypropylene (PP) Isplen PP090 62M by Repsol-YPF (Tarragona, Spain) was kindly supplied by its producer and used as the polymeric matrix. The use of a coupling agent was proposed in order to prevent chemical incompatibilities between the hydrophilic reinforcements and the hydrophobic matrix. Epolene G3015 polypropylene functionalized with maleic anhydride (MAPP) by Eastman Chemical Products (San Roque, Spain) was purchased for this purpose. This reactive has an acid number of 15 mg KOH/g and a Mn of 24800.

Decalin (decahydronaphthalene) was acquired from Fischer Scientific (Madrid, Spain) and had a 190 °C boiling point and 97% purity. This reagent was used to dissolve the PP matrix in the fiber extraction from composites.

**Figure 1:**
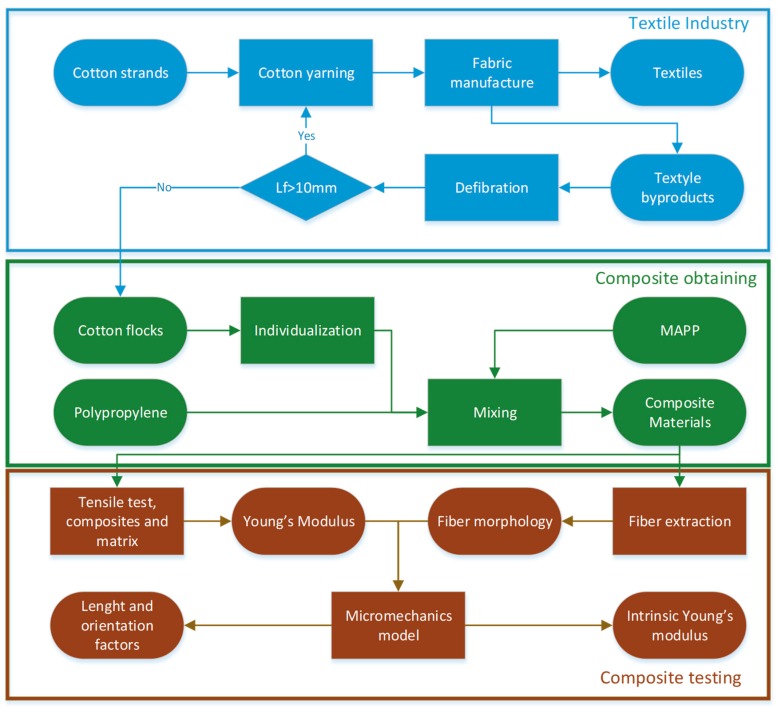
Workflow of the research, including the production of cotton flock byproducts, composite mixing and material testing.

Figure 1 shows the workflow for the research, from the production of cotton flocks by the textile industry to the measurement and evaluation of the mechanical properties.

### 2.2. Cotton Flocks Treatment and Composites Preparation

The cotton residues were passed through a blade mill in order to individualize the entangled fibers. The mill was provided with a 1mm sieve to obtain cotton fibers able to attain a good dispersion. These CFs were mixed with the PP in a Brabender Plastograph kinetic mixer by Brabender^®^ (Duisburg, Germany). The coupled composites added a 6 wt% of MAPP at the same time than the other phases. The process took 10 min, at 185 °C and at a speed of 80 rpm. Coupled and uncoupled composites with CF contents ranging from 20 to 50 wt% were prepared. The obtained blends were cut down to 8 mm pellets able to be mold injected. These pellets were stored for 24 h in an oven at 80 °C to eliminate the humidity.

### 2.3. Composite and Standard Specimen Preparation

The composite pellets were injection molded in the shape of standard dog bone specimens, in agreement with ASTM D638. The injection molding machine was a Meteor-40 by Mateu & Solé (Barcelona, Spain). The machine has three heating areas that were operated at 175 °C, 175 °C, and 190 °C, corresponding to the highest to the nozzle. The injection pressure was 120 kg/cm^−2^ and the maintaining pressure was 37.5 kg/cm^−2^. A steel mold with a cavity in the shape of the standard specimen was used, and at least ten specimens for every one of the composite formulations were obtained.

### 2.4. Mechanical Test

Prior to any mechanical test, the specimens were stored in a conditioning chamber by Dycometal. The stabilization of the specimens took 48 h, and the conditions were at 23 °C with 50% relative humidity.

The specimens were placed in the gauges of an Instron TM 1122 universal testing machine. The machine was fitted with a 5 Kn load cell. The test was performed in agreement with ASTM D790 standard. An extensometer MFA2 was used to measure the strains with addequate precision. The measurements were the result of testing at least 5 samples for every composite formulation.

### 2.5. Morphologic Analysis of the Reinforcements

Some micromechanics models use the morphology of the reinforcements as input. As soon as the literature accepts that the morphology of such reinforcements changes noticeably during composite preparation, the study was performed to reinforcements extracted from the polymeric matrix. The extraction was obtained by matrix solubilization using a Soxhlet apparatus and using Decalin as a solvent. Composite material pieces approximately 10 × 10mm were placed inside a cellulose filter into the Soxhlet equipment. The process lasted 24 h until the matrix was totally dissolved. Then, the fibers were rinsed with acetone and distilled water.

The morphology of the fibers was measured in a FS-300 Kajanni analyzer. The equipment measured from 2500 to 3000 fibers and returned a fiber length distribution, mean length, and diameter and the percentage of fines (fibers shorter than 70 µm).

## 3. Results and Discussion

### 3.1. Young’s Modulus of the Composites

Table 1 shows the Young’s moduli of the coupled and uncoupled composites (*E_t_^C^*) reinforced with CF contents ranging from 20 to 50 wt%. The table also shows the tensile strength of the composites (*σ_t_^C^*), the percentage of reinforcement in weight (*W^F^*), and its volume fraction (*V^F^*).

It was found that the use of a coupling agent had a low effect on the Young’s modulus of the composites. In fact, an ANOVA analysis (at 95% confidence rate) reveals that the differences between the Young’s moduli of the composites with the same percentage of reinforcement, despite adding or not adding a coupling agent, were not statistically relevant. This result was expected as it has been reported previously in the literature [5,32]. The same materials revealed totally different behaviors in the case of the tensile strength, where the presence of MAPP considerably increased the strength of the materials [7,20]. Thus, while the coupling agent has a noticeable effect on the tensile strength of the composites, its impact is not statistically relevant in the case of the Young’s modulus. Some authors prefer to state that the strength of the interphase between the matrix and the reinforcements has a limited impact on the stiffness of the composites [6,11]. Other authors prefer to justify the differences on the methods used to evaluate the strength and the modulus. While the strength is measured at the maximum strain, where the interphase has been fully put to test, the Young’s modulus is measured at low strains [32].

**Figure 2:**
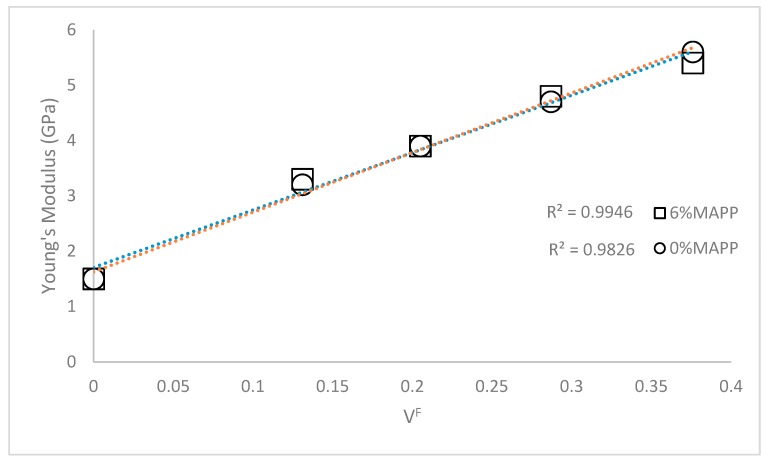
Young’s modulus of the coupled and uncoupled CF-PP composites against reinforcement content.

The Young’s modulus of a semi-aligned short fiber reinforced composite is mainly affected by the properties of the phases and its contents, the morphology of the reinforcement, its mean orientation, and its grade of dispersion. In the case of a correctly dispersed reinforcement, the increase of the Young’s modulus against reinforcement content was expected to be linear (Figure 2) [32].

Both, coupled and uncoupled composites showed a linear evolution of its Young’s moduli against CF content. Thus, a proper dispersion of the reinforcement was assumed. Nonetheless, the higher the percentage of reinforcement the harder it becomes to obtain a good or proper dispersion. In the case of the coupled composite at 50 wt% of CF, the Young’s modulus seems to start to decrease under the regression line. Besides, CF incorporates a textile dye that seems to increase the strength of the interphase and ease the dispersion at low reinforcement rates. Nonetheless, it was impossible to corroborate this evolution due to the impossibility of preparing materials with higher reinforcement contents that are still able to be mold injected. From now on, and due to the equivalence between coupled and uncoupled CF-based composites, the analysis will be referring to the coupled materials.

The literature shows multiple studies on the evolution of the Young’s modulus against the fiber contents. We have chosen stone groundwood fibers (SGW), commonly used for papermaking, hemp strands (HS), as a byproduct of agroforestry, old newspaper fibers (ONPF), as recycled fibers, and glass fibers (GF) as an industrial commodity and the most commonly used reinforcement [11,14,33]. Table 2 shows the Young’s moduli of SGW, HS, ONPF and GF reinforced PP composites.

The Young’s moduli of natural fiber reinforced polypropylene composites are similar, with slight advantages for those reinforced with strands, especially at high reinforcement contents. Cotton fibers showed Young’s moduli as superior to SGW and ONPF, and in line with the other strands. Nonetheless, cotton fibers are recycled and a byproduct of the textile industry, while hemp strands can be considered virgin materials. ONPF are recycled fibers that come from the disintegration of used newspaper. The Young’s moduli of ONPF and SGW based composites are very similar, showing that recovering the fibers from the paper had little effect on the stiffening potential of the reinforcements. Moreover, CF showed higher Young’s moduli than ONPF based composites. All the natural-based composites showed a linear evolution of their Young’s moduli against fiber contents, but different slopes on their regression lines.

On the other hand, GF-based materials showed noticeably higher Young’s moduli than natural fiber-based composites. At the same reinforcement contents, Young’s modulus of CF-based composites is noticeably lower than GF-based ones. It was necessary to increase 20 wt% the amount of CF to obtain a Young’s moduli similar to GF.

### 3.2. Neat Contribution of the Reinforcements

Attending to the above-mentioned parameters that affect the Young’s modulus of a composite, the differences must be related with the morphology of the reinforcements, its mean orientation, or the intrinsic properties of the phases. The modified rule of mixtures (RoM) for the Young’s modulus summarizes all these parameters (Equation (1)):

(1) $$E_{t}^{C}=\eta_{e}\cdot E_{t}^{F}\cdot V^{F}+\left(1-V^{F}\right)\cdot E_{t}^{M}$$

where *E_t_^C^*, *E_t_^F^*, and *E_t_^M^* are the Young’s moduli of the composite, reinforcement, and matrix, respectively. *V^F^* represents the reinforcement volume fraction, and *ƞ_e_* is a modulus efficiency factor that equalizes the contribution of the reinforcements to the Young’s modulus of the composite. This efficiency factor is seldom presented as a length efficiency factor times an orientation efficiency factor (*ƞ_e_= ƞ_l_· ƞ_o_*). At the exception of the intrinsic Young’s modulus of the reinforcements and the modulus efficiency factor, the rest of the values can be easily obtained during the tensile test of the composites. Clearly, the RoM can only be used if the Young’s modulus of the composite evolves linearly against reinforcement content.

In any case, the neat contribution of the reinforcements to the Young’s modulus of the composite is represented by *ƞ_e_·E_t_^F^* in the RoM. Thus, the RoM can be rearranged to account for such neat contribution as:

(2) $$\eta_{e}\cdot E_{t}^{F}=\frac{E_{t}^{C}-\left(1-V^{F}\right)\cdot E_{t}^{M}}{V^{F}}$$

**Figure 3:**
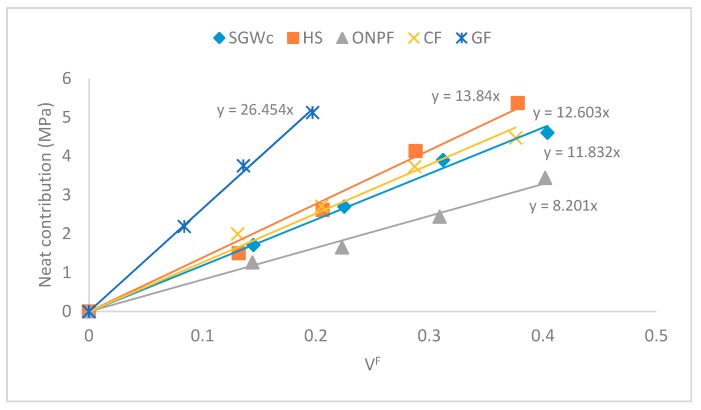
Neat contribution of the reinforcements to the Young’s modulus of the polymers.

Then, if the neat contribution is represented against the reinforcement volume fraction, a regression line is obtained, and the slope of such a line has been referred to in the literature as a fiber tensile modulus factor (FTMF) [6,32]. This factor can be used as a measure of the stiffening capabilities of a reinforcement. Figure 3 shows the FTMF for different fibers as polypropylene reinforcement.

The FTMF of CF was between HS and SGW. This value ensures good stiffening abilities for CF as PP reinforcement because the literature shows possible applications for materials with similar FTMF for building or product design purposes [34,35]. Moreover, some researchers used an ONPF-based composite to substitute a GF-based one [36]. On the other hand, GF showed higher stiffening capabilities than the rest of the reinforcements. This is not a surprise, having in account that GF is a man-made material with more stable intrinsic properties and a regular morphology. The FTMF of the reinforcements shows a similar behavior than the Young’s moduli of its composites. Thus, the differences between such moduli seem to be focused on the neat contribution of the fibers, specifically, the intrinsic Young’s modulus of the reinforcement and the modulus efficiency factor. In order to analyze such differences, the researchers propose a micromechanics analysis.

### 3.3. Micromechanics Analysis of the Young’s Modulus

The RoM (Equation (1)) shows two unknowns that coincide with the neat contribution of the fibers: *ƞ_e_·E_t_^F^*. While it is possible to measure the intrinsic Young’s modulus of the fibers, and more so in the case of the strands, some authors defend the use of micromechanics methods as an alternative [11,37,38]. In addition, a high number of experiments are necessary due to the foreseeable standard deviations of the mechanical properties of natural fiber reinforcements. Thus, the Hirsh model was proposed as a means to evaluate the intrinsic Young’s modulus of CF.

(3) $$E_{t}^{C}=\beta \cdot (E_{t}^{F}\cdot V^{F}+E_{t}^{M}\left(1-V^{F}\right)+\left(1-\beta \right)\frac{E_{t}^{F}\cdot E_{t}^{M}}{E_{t}^{F}\cdot V^{F}+E_{t}^{m}\left(1-V^{F}\right)}$$

where *β* is a parameter that modules the stress transference between both phases of the composite material. In the case of semi-aligned short fibers reinforced composites *β* has a value of 0.4 [14]. Table 3 shows the micromechanical parameters obtained after the analysis.

The mean intrinsic Young’s modulus of CF was found to be 27.87 ± 2.63 GPa, similar to HS, with a value of 26.8 GPa [11]. This coincidence agrees with the already similarities found in the neat contributions of such fibers (Figure 3). Nonetheless, the computed intrinsic Young’s modulus of CF contrasts heavily with the neatly inferior values found in the literature. Some authors place this intrinsic Young’s modulus in the range from 5 to 13 GPa [26,27,28]. Using such values with the RoM is not possible to reach the obtained experimental values without using modulus efficiency factors outside the usual range.

On the other hand, the value for CF is noticeably higher than the value obtained for SGW and ONPF, 21.2 ± 1.9 and 22.8 ± 1.8 GPa, respectively [14,39]. This also agrees with the neat contributions of such fibers. Similarly, GF showed an intrinsic Young’s modulus of 76 GPa, justifying the differences obtained in the Young’s modulus of its composites [11,40].

Consequently, the intrinsic Young’s moduli of the different reinforcements affected heavily the Young’s moduli of its composites. Nonetheless, CF showed a higher intrinsic Young’s modulus than HS, but HS-based composites showed higher Young’s moduli, at the same reinforcement contents than CF-based composites. Thus, the differences are expected to be found in the modulus efficiency factor (Table 3).

The values for the modulus efficiency factor were obtained by using all the experimental data (Table 1) and the mean intrinsic Young’s modulus of CF (Table 3). The mean value was found to be 0.47 ± 0.03. The value is inside the usual range of values, between 0.45 and 0.56 for such factor [11,12,14,40]. Nonetheless, it is worth noting that the obtained value is in the lower half bound of the expected values. Thus, presumably, CF based composites have not taken advantage of the stiffening capabilities of CF. Particular values decrease when the CF contents increase (Table 3). The composite with a 20 wt% of CF exhibits a modulus efficiency factor higher than the other CF-based composites, and also a higher intrinsic Young’s modulus, indicating a higher yield on the stiffening capabilities of CF. The reasons must be found on the mean orientation of the fibers, the morphology of the reinforcements or its dispersion.

In the case of HS the modulus efficiency factor was evaluated at 0.50 ± 0.02. This value is higher than CF and can compensate the difference between the intrinsic Young’s moduli of the reinforcements and justify the higher moduli of the HS-based composites. In the case of ONPF, the value of *ƞ_e_* was evaluated at 0.49 ± 0.04, a value similar to HS. Finally, SGW showed the highest values for *ƞ_e_*, with a mean of 0.56 ± 0.02.

In order to find the impact of the morphology and the mean orientation of CF, the morphology and orientation efficiency factors were computed. The length efficiency factor was calculated according to Cox-Krenchel’s model (Equations (4) and (5)) [41]:

(4) $$\eta_{l}=1-\frac{tanh\left(\frac{\mu \cdot L^{F}}{2}\right)}{\left(\frac{\mu \cdot L^{F}}{2}\right)}$$

with

(5) $$\mu =\frac{1}{r^{F}}\sqrt{\frac{E_{t}^{M}}{E_{t}^{F}\cdot \left(1-\upsilon \right)\cdot Ln\left(\sqrt{\pi /4\cdot V^{F}}\right)}}$$

where *L^F^* and *r^F^* are the reinforcement mean weighed length and radius, respectively. The Poisson’s ratio of the matrix is represented by *ν* and *µ* is a coefficient of the stress concentration rate at the end of the fibers. The Poisson ratio was 0.36, as found in the literature [22]. The orientation factor *η_o_* was obtained from *η_o_* = *η_l_*/*η_e_*. Table 3 shows the obtained values.

The length efficiency factor remained almost the same for all the composite formulations, with a mean value of 0.89 ± 0.01. Usually this factor decreases when the percentage of reinforcement increases [5]. This is due to the changes in the mean length of the reinforcements during compounding, when reinforcements are exposed to attrition phenomena and tend to break. There is a decrease in the mean length of such reinforcements as the reinforcement content increases [32]. Thus, these changes are expected to affect the Young’s moduli of the composites. In the case of CF based composites, the impact of the morphology of the fibers seems to little impact the Young’s modulus, although the reinforcements decreased their mean length from 293 to 185 µm [7]. This hypothesis will be put to test later on by applying a different micromechanics model.

On the other hand, the orientation efficiency factor clearly changed with the amount of reinforcement (Table 3). This factor showed a mean value of 0.53 ± 0.04 and ranged from 0.58 to 0.49. Usually, the orientation efficiency factor is more stable than the length efficiency factor, because the mean orientation of the fibers is heavily impacted by the geometry of the injection mold and the parameters used during the mold injection [42,43].

Fukuda and Kawata [44] studied the tensile modulus of short fiber reinforced thermoplastics, and the orientation of the fibers inside the composites. The authors proposed different fiber distributions, but based on the literature, a rectangular distribution (square packing) renders adequate results for short fiber semi-aligned reinforced composites [32,45]. The authors present an equation that computes the orientation efficiency factor from a mean orientation angle (α):

(6) $$\eta_{o}=\frac{sin\left(\alpha \right)}{\alpha }\left(\frac{3-\upsilon }{4}\frac{sin\left(\alpha \right)}{\alpha }+\frac{1-\upsilon }{4}\frac{sin\left(3\alpha \right)}{3\alpha }\right)$$

Equation (6) was used to compute the mean orientation angles of the reinforcements (Table 3). The mean orientation angle was found to be 53.3 ± 3.3°. This value is in line with other natural fiber reinforced composites, thought in the upper bounds, meaning that the reinforcements are les oriented than the expected. It must be stressed that the orientation decreased with the amount of reinforcement, from 48.8° for the composite containing 20 wt% of CF to 56.2° for the composite containing 50 wt%. In other cases, it was found that the composites with higher fiber contents showed higher orientations [11]. In these studies, it was assumed that the shorter fibers were easily aligned than the longer ones. Nonetheless, the RoM does not incorporate a factor taking in account the dispersion of the fibers, and as has been previously commented, the authors suspect that the dispersion of the composites at high-reinforcement contents was improvable.

### 3.4. Effect of the Morphology of the Reinforcements

While the rule of mixtures (Equation (1)) and Hirsch’s equation (Equation (3)) are elegant models that allow predicting the Young’s modulus of a composite from a variety of parameters, they do not incorporate any morphological property of the reinforcements. The morphology of the reinforcements is known to greatly impact the mechanical properties of a composite, especially the ratio between its mean length and diameter, known as aspect ratio [12,46,47]. Thus, the authors propose using the Tsai and Pagano model (Equation (7)) in combination with Halpin and Tsai equations (Equations (8)–(10)) to evaluate a theoretical Young’s modulus of the composites.

In agreement with Tsai and Pagano model, the stiffness in the fiber direction is given by:

(7) $$E_{t}^{C}=\frac{3}{8}E^{11}+\frac{5}{8}E^{22}$$

where, *E*^11^ and *E*^22^ are the longitudinal and transversal elastic modulus, calculated by the Halpin–Tsai equations [11]:

(8) $$E^{11}=\frac{1+2\left(L^{F}/2r^{F}\right)\cdot \lambda_{l}V^{F}}{1-\lambda_{l}V^{F}}E_{t}^{M}$$

with

(9) $$\lambda_{l}=\frac{\left(E_{t}^{F}/E_{t}^{M}\right)-1}{\left(E_{t}^{F}/E_{t}^{M}\right)+2\left(L^{F}/2r^{F}\right)}$$

and:

(10) $$E^{22}=\frac{1+2\lambda_{t}V^{F}}{1-\lambda_{t}V^{F}}E_{t}^{M}$$

with

(11) $$\lambda_{t}=\frac{\left(E_{t}^{F}/E_{t}^{M}\right)-1}{\left(E_{t}^{F}/E_{t}^{M}\right)+2}$$

Table 4 presents the obtained values. The values obtained by using the micromechanics model show a good alignment with the experimental values, especially at reinforcement contents higher than 20 wt%. In these cases, the error goes to a maximum of 5.6%, which is assumable when modeling a mechanical property of a natural fiber reinforcement composite. In fact, the standard deviation of the experimental values is higher than this maximum of 5.6% (Table 1). The higher errors found for the composite with a 20 wt% of CF can be explained by the intrinsic Young’s modulus derived from these composites (Table 2). The value of such a parameter is 11.5% higher than the mean value. If the 31.48 GPa value is used as input for the Tsai and Pagano model, the theoretical Young’s modulus increases to 3.02 GPa. This value is more similar to the value obtained experimentally. Moreover, the authors blame the differences between the experimental and theoretical values to deviations from a totally linear evolution of the Young’s moduli against reinforcement contents—due to possible auto entanglements between the CF—and thus showing a slightly worse dispersion of the CF.

**Figure 4:**
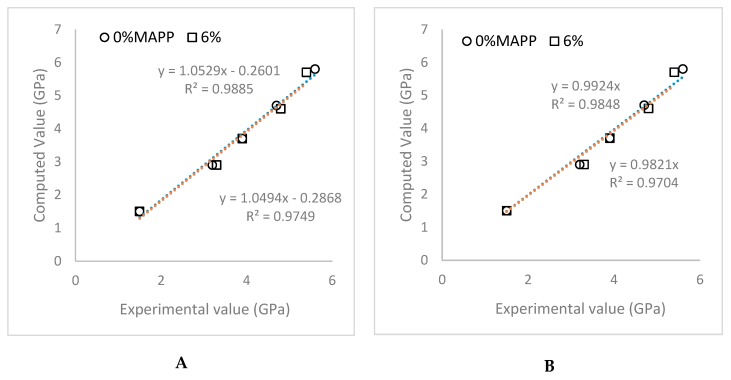
Correlation between the experimental Young’s moduli of the composites and the computed ones by using the Tsai and Pagano model in combination with Halpin and Tsai equations: (**A**) Unweighted correlation; (**B**) correlation line adding the condition of such line going through the origin.

The obtained values were plotted against the experimental ones to find the grade of correlation between both values (Figure 4).

The regression lines for the uncoupled and coupled composites using the experimental and theoretic values (Figure 4A) show 1.05 slopes, with a high correlation. This slope shows a high coincidence between the computes and experimental values. A second regression line was proposed, passing thorough the origin (Figure 4B). In this case the slopes were found to be 0.99 and 0.98 for the uncoupled and coupled composite, respectively. As mentioned above, these results prove the accuracy of the values predicted from the model. The values also prove the impact of the morphology of the reinforcements in the Young’s moduli of the polymers, as Tsai and Pagano model uses these values as the input.

## 4. Conclusions

Composite materials with CF as reinforcement and PP as the matrix were formulated with 20 to 50 wt% CF contents. Two batches were prepared, one including a 6 wt% of coupling agent and another without.

The Young’s moduli of the composites were little impacted by the presence of coupling agents and thus by the strength of the interphase. Thus, for applications where stiffness is paramount, uncoupled composites can be used with the same response under load as the coupled composites. On the other hand, in the case of semi-structural uses, the coupled composites ensure a higher tensile strength and similar deformations under load than the uncoupled ones.

The Young’s moduli of the composites were similar to those obtained for natural strands reinforced composites and higher than wood fibers reinforced composites. The presence of a textile dye in the CF decreased its hydrophilicity but also seemed to increase the difficulty in obtaining a good dispersion of the fibers inside the composite, and the decrease in the quality of the dispersion decreased the stiffening yield of CF.

A value of the intrinsic Young’s modulus of CF was obtained by using Hirsh’s model. The mean value of the modulus was found to be 27.87 GPa, lower than other strands. Nonetheless, this value doubles the values previously published. The value obtained for the CF in a composite with 20 wt% of reinforcement reached 31.48 GPa, a value more proper to other natural strands like hemp.

The micromechanics properties of the Young’s modulus of the composites showed the effect of the orientation and the morphology of the fibers. Nonetheless, the authors found some deviations from a linear behavior of the Young’s modulus against CF contents. The authors assume that the dispersion of the fibers can be improved to increase the Young’s moduli of the composites with high rates of reinforcement. Nonetheless, further research is needed to prove this point.

This paper shows the opportunity of recovering textile cotton fibers, which are useless for the textile industry, to obtain composite materials able to replace glass fiber reinforced materials. In doing so, the dumping and incinerating of such fibers is avoided and the value chain of the textile industry is widened.

## Figures and Tables

**Table 1:** Young’s modulus and tensile strength of the cotton fiber (CF)/polypropylene (PP) composites.

		0%MAPP		6%MAPP	
*W^F^*	*V^F^*	*E_t_^C^* (GPa)	*σ_t_^C^* (MPa)	*E_t_^C^* (GPa)	*σ_t_^C^* (MPa)
0	0	1.5 ± 0.1	27.6 ± 0.5	1.5 ± 0.1	27.6 ± 0.5
20%	0.131	3.2 ± 0.1	35.0 ± 0.5	3.3 ± 0.1	41.7 ± 0.7
30%	0.205	3.9 ± 0.2	38.2 ± 0.8	3.9 ± 0.1	47.1 ±0.7
40%	0.287	4.7 ± 0.2	41.7 ± 0.8	4.8 ± 0.2	53.6 ± 1.0
50%	0.376	5.6 ± 0.2	45.4 ± 1.1	5.4 ± 0.2	58.3 ± 1.2

**Table 2:** Young’s moduli of stone groundwood, hemp strands, and glass fiber reinforced polypropylene coupled composites.

	SGW	HS	ONPF	GF
20%	2.7 ± 0.1	2.8 ± 0.1	2.8 ± 0.1	4.1 ± 0.1
30%	3.5 ± 0.1	3.8 ± 0.1	3.8 ± 0.1	5.7 ± 0.1
40%	4.3 ± 0.1	5.2 ± 0.1	4.2 ± 0.1	7.7 ± 0.1
50%	5.2 ± 0.1	6.3 ± 0.1	5.3 ± 0.1	-

**Table 3:** Micromechanics of the Young’s moduli of CF reinforced polypropylene coupled composites.

*V^F^*	*E_t_^F^* (GPa)	*ƞ_e_*	*ƞ_l_*	*ƞ_o_*	*α_o_*
0.131	31.48	0.52	0.89	0.58	48.8
0.205	28.06	0.47	0.89	0.53	53.3
0.287	26.48	0.45	0.89	0.51	55.1
0.376	25.46	0.45	0.90	0.49	56.2
Mean	27.87 ± 2.63	0.47 ± 0.03	0.89 ± 0.01	0.53 ± 0.04	53.3 ± 3.3

**Table 4:** Theoretical Young’s moduli of the composites computed by using the Tsai and Pagano model in combination with Halpin andTsai equations.

	Experimental		Tsai-Pagano		Error (GPa)		Error (%)	
*V^F^*	0% MAPP	6% MAPP	0% MAPP	6% MAPP	0% MAPP	6% MAPP	0% MAPP	6% MAPP
0.131	3.2	3.3	2.9	2.9	0.3	0.4	9.4	12.1
0.205	3.9	3.9	3.7	3.7	0.2	0.2	5.1	5.1
0.287	4.7	4.8	4.7	4.6	0	0.2	0	4.2
0.376	5.6	5.4	5.8	5.7	–0.2	–0.3	–3.6	–5.6
